# Food Safety of Consuming Black Soldier Fly (*Hermetia illucens*) Larvae: Microbial, Heavy Metal and Cross-Reactive Allergen Risks

**DOI:** 10.3390/foods10081934

**Published:** 2021-08-20

**Authors:** Leah W. Bessa, Elsje Pieterse, Jeannine Marais, Karim Dhanani, Louwrens C. Hoffman

**Affiliations:** 1 Department of Animal Sciences, University of Stellenbosch, Stellenbosch 7600, South Africa; leahbessa@gmail.com (L.W.B.); elsjep@sun.ac.za (E.P.); 2 Department of Food Science, Stellenbosch University, Private Bag X1, Matieland 7602, South Africa; jeanninemarais@sun.ac.za; 3 The Woodmill Office 11, 1st Floor, Vredenburg Road, Stellenbosch 7602, South Africa; karim@factssa.com; 4 Centre for Nutrition and Food Sciences, Queensland Alliance for Agriculture and Food Innovation, University of Queensland, Coopers Plains, QLD 4108, Australia

**Keywords:** edible insects, black soldier fly larvae, allergenic peptides, microbiological safety, heavy metals

## Abstract

Black soldier fly (*Hermetia illucens*) larvae (BSFL) are a promising, sustainable source of nutrients, however, there is limited knowledge regarding the food safety of consuming BSFL. This study determined the safety of consuming BSFL for direct human consumption in terms of microbial, heavy metal and allergen content. Microbial counts were determined using ISO (International Organization for Standardization) methods, heavy metals were determined using inductively coupled plasma mass spectrometry and allergens were determined via Orbitrap mass spectrometry and ELISA (enzyme-linked immunosorbent assay) kits. Feed and killing method influenced the presence of *Bacillus cereus* (*p* = 0.011), and only the killing method influenced *Escherichia coli* (*p* < 0.00) and total viable count (TVC) (*p* < 0.00). Blanching resulted in a 3-log reduction in *E. coli* and a 3.4 log reduction in the TVC counts. *Salmonella* spp. and *Listeria* spp. were not detected in the BSFL samples. Heavy metals were detected although they were below maximum legal limits. Cross-reactive allergens, tropomyosin and arginine kinase, were detected in the BSFL samples, although the clinical significance requires research. The feed fed to the BSFL and blanching were found to influence the safety of consuming BSFL, highlighting the importance of incorporating sufficient decontamination steps, such as blanching, to ensure food safety.

## 1. Introduction

Black soldier fly larvae (BSFL) hold the promise of being a sustainable, nutritious, economically viable food source, however, there are still some challenges that lay ahead before BSFL could be considered a sufficiently safe food source for human consumption [1]. One study has indicated consumers’ willingness to consume BSFL in a processed form [2], but the food safety elements of BSFL remain largely unknown, since BSFL have been used almost exclusively for animal feed purposes. There is, however, substantial research regarding the safety of using BSFL for use in animal feed, with data on BSFL reared on many different waste substrates, ranging from spent grains to organic waste from crop agriculture, which could also be useful for human food purposes [3,4,5,6,7,8,9].

High microbial contents have been identified across edible insect species, with pathogenic bacteria such as *Escherichia coli*, *Bacillus cereus* and *Staphylococcus aureus* being of particular concern [10]. Factors such as feed, farming environment and post harvesting processes (blanching) have an influence on the microbial load [11,12], with blanching being a particularly effective tool in reducing the overall microbial load of insects intended for human consumption [12,13]. As with any food product, storage conditions are important in keeping insect products safe. Shelf-life studies have demonstrated a rapid increase in bacterial growth when insects (wet) were stored at room temperature (21–24 °C), while bacterial growth slowed drastically when stored in refrigerated environments (1–2 °C) [11,12]. Of all storage conditions, drying has been found to be the best method for extending shelf-life of insects in both refrigerated and room temperature conditions [12].

When considering BSFL specifically for human food, growing BSFL on ‘waste’ can raise concerns for their use in food because of the potential contaminants that could accumulate from the feed. Regarding microbial contamination, studies have shown BSFL’s ability to significantly reduce Enterobacteriaceae colonies and *Salmonella* spp. in a variety of different feed sources ranging from manure [2,14] to fecal matter [15]. This is because in nature BSFL are innately decomposers and they have a multitude of mechanisms in place to reduce the microbial load in their feed. Due to the high pH of their gut (9.3), gut enzymatic reactions and competitive gut bacteria, some bacterial species (e.g., *E. coli*, *Salmonella* spp.) from the feed are not able to survive [6].

Another contaminant of concern is the heavy metal content. Studies have shown that BSFL bioaccumulate certain heavy metals from their feed, with heavy metals such as cadmium, copper, lead and mercury being of particular concern due the severe health risks associated with their consumption [8,16,17,18,19,20]. Mercury for example can have bioaccumulation factors ranging from 1.6 [17] to 4.5 [18] in BSFL, and cadmium, which is highly accumulative in BSFL, has bioaccumulation factors of between 2.5 to 9.5 [18]. It is therefore important to understand the heavy metal risks associated with the waste stream intended to feed the larvae on, to mitigate the risk to human health. Selected heavy metals also negatively affect the growth of BSFL, by causing an imbalance with their gut microbiome, therefore it is equally important to consider the heavy metal content for larval health [21].

Allergens from BSFL are another safety concern that need to be fully investigated and understood prior to introducing BSFL as a commercial food source [22,23,24]. Cross-reactive allergens such as tropomyosin, arginine kinase and myosin have been identified in both BSFL and crustaceans and are known allergens for Arthropoda in the Allergen Nomenclature database (http://www.allergen.org/index.php, accessed on 30 July 2021). Cross-reactive allergens increase the likelihood of allergic reactions occurring in consumers who would consume BSFL that are allergic to crustaceans [24,25]. Examples of cross-relatively between crustaceans and edible insect species have been documented with examples such as: tropomyosin being a cross-reactive allergen in both mites and in shrimp [25]; arginine kinase in prawn species and in silkworm larvae (*Bombyx mori*) [26], the lesser mealworm (*Alphitobius diaperinus*) [24], field cricket (*Gryllus bimaculatus*) and the Indian meal moth (*Plodia interpuntella*). To date, few studies have investigated allergens in BSFL, and the ones that did report conflicting results. In one study arginine kinase was not identified as a major cross-reactive allergen in BSFL [24], although in another study arginine kinase was identified as a major cross-reactive allergen in BSFL protein extract [27]. This just highlights the need for more research into BSFL allergens, as there is a huge risk of consumers who are allergic to crustaceans being allergic to BSFL as well, which in turn will influence the labelling of BSFL products for human consumption [23]. Processing conditions can also affect the allergenic proteins found in BSFL, as previous studies demonstrated that allergenic proteins in other edible insects were unaffected by heating and digestion [22,28], whereas others were reduced or inactivated completely [29,30,31,32]. In some cases, heating even caused proteins to have more of an allergic reaction [29]. Once again, this information calls for further validation through a wider body of research into BSFL allergens for safe human consumption [1].

Currently the regulations in the EU do not support the use of BSFL in human food, and there are only guidelines regarding the safe use of mealworms for human consumption [29]. Prior to that, food safety reports indicating the microbial risks associated with edible insect species based their suggested limits on EU safety regulations for meat and seafood, due to the high moisture and nutrient content of the insects [33,34]. While the regulations for meat and seafood can offer guidelines regarding microbial contaminants, it is important that further research goes into understanding the microbial risks associated specifically with BSFL reared on a variety of feeds that could cause concerns in a food application.

Hazard analysis and critical control points (HACCP) is a systematic tool that identifies and mitigates hazards in food systems, and it is strongly recommended for implementation in insect processing plants as a preventative measure to mitigate food safety risks that could arise when processing these novel foods [35]. The challenge regarding the implementation, is that the HACCP principles require in depth knowledge about the potential hazards associated with the products and its processes. Even though there is an increasing body of information on the potential risks of using BSFL as human food, far more reliable and consistent evidence is required to develop a robust HACCP process with successful outcomes for BSFL processing, especially if they are fed on by-products/waste streams [35].

The safety of consuming BSFL is also an important factor in gaining consumers acceptance and it is imperative to investigate the larvae from a food safety perspective before fully advocating its consumption [36,37,38]. Furthermore, while its role in recycling organic matter is one of its biggest advantages, it is also one of the biggest concerns regarding BSFL as a food source, due to the potential contaminants the larvae may accumulate in the process of reducing waste. It is therefore presently suggested that BSFL be reared on clean ‘waste’ such as spent grain from brewing or by-products of food production to mitigate some of the proposed risks such as microbial contamination and heavy metal uptake [5,36]. The aim of this study was therefore to determine whether BSFL grown on three different feeds (by-products of food production) and killed using two different methods (freezing and blanching) would be considered safe to eat according to EU food safety standards.

## 2. Materials and Methods

### 2.1. Experimental Design

The experimental design consisted of BSFL grown on three different feeds, with six independent batches of each feed to account for natural variation. Analyses were conducted to compare the effect of feed and killing method (blanched/frozen) on the safety of the larvae.

### 2.2. Rearing and Sample Preparation

Black soldier fly larvae were obtained from MaltEnto; a commercial BSFL farm (19 Moody Ave, Epping, Cape Town, South Africa). Three different, isolipidic feeds were used to grow the larvae and consisted of a broiler feed-base diet and two by-product feeds (Table 1). Trays (800 mm × 300 mm × 400 mm) were populated with BSFL, and feed (Table 1) was added to the trays (70 mm deep). The growing environment was monitored and maintained at 27.0 ± 1.0 °C and 70% relative humidity. The larvae were randomly harvested from six different batch containers (1 kg in total) (to allow for natural variation) on day 11 (from hatching) by sieving them out of the feed and fasting them for 6 h [39]. The larvae were then killed using two different methods: freezing (−20 °C) for 24 h; and blanching at 100 °C for 60 s. The BSFL were analyzed to determine if there were any contamination from the feed by performing microbial and heavy metal analysis on the feed and larvae fed the three different feeds. The BSFL samples were also analyzed for the presence of any cross-reactive crustacean allergens, as well as the relative abundance of the latter between killing methods.

### 2.3. Microbiological Tests

Black solider fly larvae were rinsed with a saline solution, followed by rinsing again with distilled water to remove any dirt, or contamination that would have resulted from the farming environment.

For each test, 10 g of the raw insect was added to 90 mL of autoclaved Physiological Salt Solution (PSS) in a stomacher bag, and then homogenized for 2 min (Seward stomacher 400). A dilutions series (10^−1^ to 10^−6^) of the BSFL-PSS solution was prepared and used for each test. All agar and solutions were prepared according to manufacturer’s specifications (Merck, Johannesburg, South Africa) and all tests were carried out using International Organization for Standardization (ISO) approved methods. Total viable count (TVC) was determined by spread plate on Tryptic Soy Agar (TSA) (Merck, South Africa), and then incubated at 37 °C for 48 h [40,41]. Aerobic endospores were tested by heating the sample to 75 °C for 20 min, and then spread plating it on TSA agar (Merck, South Africa) and incubating at 35 °C for 48 h according to ISO 21871 [42]. Enumeration of *E. coli* was tested by the spread plate technique on Violet Red Bile Agar (VRBA) (Merck, South Africa) and incubated at 30 °C for 24 h according to ISO 21528-2 [43].

The presence of *Listeria monocytogenes* was tested for using a Fraser and half Fraser broth according to ISO 11290-2:1998 + A1 [44]. The presence of *Salmonella* was tested for in four successive stages according to ISO 6579:2002 + A1. First, 25 g of larvae from each killing method was added to Buffered Peptone Water (BPW) and incubated at 35 °C for 24 h. Thereafter, 0.1 mL of the incubated samples were transferred to 10 mL of *Salmonella* enrichment broth (Merck, South Africa) and incubated for 42 °C for 24 h, and then for a further 24 h at 35 °C [45]. The inoculated *Salmonella* enrichment broth samples were streaked out on Xylose Lysine Deoxycholate (XLD) (Merck, South Africa) agar plates and incubated at 35 °C for 24 h [45]. All microbiological plates were inspected for colonies after incubation via observational counting.

### 2.4. Heavy Metals

The concentration of 10 metals [Al (Aluminum), As (Arsenic), Cd (Cadmium), Cr (Chromium), Fe (Iron), Hg (Mercury), Mn (Manganese), Pb (Lead), Sn (Tin), Zn (Zinc)] were analyzed via Inductively Coupled Plasma Mass Spectrometry (ICP-MS) at the Central Analytical Facility (CAF) (Stellenbosch University, Lombardi Building, Room 3016, Cnr Neethling and Victoria Street, Stellenbosch, 7600). Insect and feed samples were prepared by freezing them at −20 °C after which they were lyophilized in a freeze drier (Christ Aplha 1–2 LSC basic, Osterode am Harz, Germany). Dried samples were then ground into a powder using a laboratory grinder for 20 s prior to digestion (Foss Knifetec 1095 sample mill, Hilleroed, Denmark). Digestion took place using a pressurized microwave (CEM Mars 5 Digestion Oven, Torrance, CA, USA) acid extractable digestion (ultra-pure HNO_3_ and HCl). Once the sample was cooled, the extractant was made up to 50 mL with milliQ water, then analyzed on an Agilent 7900 quadrupole ICP-MS for the previously mentioned elements. Samples were introduced via a 0.4 mL/min micromist nebulizer into a Peltier cooled spray chamber (2 °C). The instrument was optimized in high matrix introduction (HMI) mode, where both samples and standards were diluted with argon gas to minimize the matrix load to the analyzer. The formation of oxide was less than 0.3%. All elements were measured in He-collision mode, using an internal standard solution containing Germanium (Ge), Indium (In), Rhodium (Rh), Scandium (Sc) and Yttrium (Y) that was introduced online to monitor instrument drift and correct for matrix differences. These internal standard solutions were matched to the analytes based on their proximity in mass and ionization potential. The instrument was calibrated using NIST traceable standards from Inorganic Ventures (Inorganic Ventures, 300 Technology Drive, Christiansburg, VA 24073, USA). The accuracy of the calibration was verified by analyzing the NIST traceable standards at high and low concentrations (De Bruyn Spectroscopic Solutions, Bryanston, South Africa) prior to analysis of the samples and after 12 samples to monitor drift. The internal standard solution recovery was in the range of 90 to 110% and the recovery for the drift monitor standards were between 95% and 105%. Triplicate measurements were carried out on each analyte, on 3 aliquots per sample.

Bioaccumulation of heavy metals was determined using the bioaccumulation factor (BAF) on a dry matter basis. A BAF > 1 indicates that the BSFL bioaccumulated the heavy metal from the feed [17].

BAF = (concentration of heavy metal organism)/(concentration of heavy metal in feed)

### 2.5. Allergen Detection

The BSFL samples were tested to determine if there was any presence of cross-reactive crustacean allergens, as well as their relative abundance when raised on the three diets and between killing methods. All analyses were performed in the laboratories at the Food and Allergy Consulting and Testing Services (FACTS SA, The Woodmill Office 11—1st Floor, Vredenburg Road, Stellenbosch, South Africa) according to the methodology described below.

#### Protein Extraction and Preparation

Protein identification was carried out using an untargeted proteomic approach using liquid chromatography mass spectrometry (LC-MS). A Thermo Scientific Ultimate 3000 RSLC (Waltham, MA, USA) equipped with a 5 mm × 300 µm C18 trap column (Thermo Scientific) and a CSH 25 cm × 75 µm 1.7 µm particle size C18 analytical column (Waters, Milford, MA, USA) was used to perform liquid chromatography. Thereafter the solvent was loaded (2% acetonitrile:water; 0.1% FA; Solvent A:2% acetonitrile:water; 0.1% FA; and Solvent B:100% acetonitrile:water) onto the trap column using loading solvent (flow rate of 2 µL/min; at 7 °C). Loading began 5 min before the sample was eluted onto the analytical column, thereafter the flow rate was adjusted to 250 nL/min and the gradient was generated as follows: 5.0–35% B over 60 min and 35–50% B over 60–75 min. Liquid chromatography was performed at 40 °C and the outflow delivered to the mass spectrometer through a stainless-steel nano-bore emitter.

Orbitrap mass spectrometry was performed using a Thermo Scientific Fusion Mass Spectrometer equipped with a Nanospray Flex ionization source (Waltham, MA, USA) and was used to match spectra to the closest scaffold. The sample was introduced through a stainless-steel emitter and the data was collected in positive mode with spray voltage set to 1.8 kV and ion transfer capillary set to 280 °C. Spectra were internally calibrated using polysiloxane ions (*m*/*z* = 445.12003 and *m*/*z* = 371.10024). Mass Spectrometer One scans were performed using the orbitrap detector (120,000 resolution) over the scan range 350–1650 with AGC target at 3, E5 and maximum injection time of 40 ms. Two acquisitions were performed using monoisotopic precursor for ion selection with charges +2 +7 (error tolerance +/− 10 ppm). Precursor ions were excluded from fragmentation for a period of 60 s thereafter precursor ions were selected for fragmentation in HCD mode using the quadrupole mass analyzer (HCD energy = 30%). Fragment ions were then detected in the orbitrap mass analyzer (30,000 resolution). The AGC target was set to 5 × 10^4^ and the maximum injection time to 80 ms. The data was acquired in centroid mode.

The mass spectrometer raw data was imported into Proteome Discoverer v1.4 (Thermo Scientific) and processed using the Sequest and Amanda algorithms which identifies peptides out of high-resolution mass spectrometry data. Database interrogation was carried out using a database created with the cRAP contaminant database (https://www.thegpm.org/crap/, accessed on 30 July 2021) concatenated with the uniprot databases (www.uniprot.org, accessed on 30 July 2021), allowing for semi-tryptic cleavage with two missed cleavages. Precursor mass tolerance was set to 10 ppm and fragment mass tolerance set to 0.02 Da. Demamidation (NQ) oxidation (M) and acetylation of protein N-terminal was allowed as dynamic modifications and thiomethyl of C as static modification. The target-decoy peptide-spectrum match (PSM) validator node was used for peptide validation. The search results were imported into Scaffold Q+ (www.proteomesoftware.com, accessed on 30 July 2021) for further validation. The HRMS dataset was concatenated with the drosophila database.

Relative quantitation data was obtained on a QTRAP 6500 LC-MS/MS system (Sciex. Darmstadt. Germany) using an IonDrive Turbo V ESI source, in positive mode coupled to a Sciex ExionLC ultra high-performance liquid chromatography (UHPLC). A Phenomenex Aeris 2.6 μm Peptide XB-C18. 100 Å (100 mm × 2.1 mm) column was used for LC separation of tryptic digests (flow rate 300 μL/min) using a three-step acetonitrile and water gradient at a full duty cycle time (20 min). The data was acquired on Analyst software (v1.7) operating in Multiple Reaction Monitoring (MRM) mode. Multiple Reaction Monitoring parameters were tuned with synthetic peptides using syringe pump injection and chromatographic runs. Quadrupole one and quadrupole three were both set to unit resolution of 0.7 ± 0.1 amu. Tryptic digests were used as control samples to survey retention time, stability, and relative intensity of the MRM transitions. The data was evaluated using Sciex OS v1.7.

### 2.6. ELISA

Enzyme-linked immunosorbent assay (ELISA) kits (RIDASCREEN^®^FAST Crustacean R7312; r-BioPharm; AEC Amersham, Cape Town, South Africa) specifically for crustacean allergens (mainly tropomyosin) were used to determine whether crustacean allergens would be identified in the BSFL samples. The ELISA kit was a sandwich enzyme immunoassay and was calibrated to whole crustaceans. ELISA kits and testing was carried out according to manufacturer’s instructions.

### 2.7. Statistical Analysis

A completely randomized split analysis of variance (ANOVA) was carried out with feed as the main plot factor and killing method as the subplot factor. Shapiro-Wilk test was conducted to test for deviation from normality, however, for these analyses the normality assumptions were not violated. For post hoc testing, the Fisher least significant difference (LSD) test was used. Correlation analyses were carried out using Pearson correlation. A 5% significance level was used. Statistical analysis was conducted using SAS software (Version 9.4; SAS Institute, Cary, NC, USA). Descriptive statistical analysis was used to show the means and standard deviations of the feeds.

## 3. Results

### 3.1. Microbiological Analysis

The interaction between feed and killing method (F-frozen and B-blanched) was the most prominent interaction for *Bacillus cereus* (*p* = 0.011), whereas killing method had a significant effect on *Escherichia coli* (*p* < 0.001) and TVC (*p* < 0.001) (Table 2). No interaction was found for *Salmonella* spp. and *Listeria* spp. as there was no colonies detected on the feed or any of the BSFL samples, therefore they are not displayed in Table 2.

The TVC and *B. cereus* of the three different feeds exceeded 5.5 log cfu/g, as did the TVC of the frozen BSFL grown on the three different feeds (Table 3). The blanched larvae had lower TVC counts (*p* < 0.05), and the TVC of the blanched larvae ranged from 2.07 log cfu/g (F1 and F3) to 2.2 log cfu/g (Table 3). The *B. cereus* counts on the frozen larvae grown on F2 were the highest at 2.2 cfu/g (*p* < 0.05) whilst the larvae grown on F1 (frozen) and F2 (blanched) had *B. cereus* counts of 2.0 log cfu/g (Table 3). Only the blanched larvae grown on F1 had lower *B. cereus* counts of 1.7 log cfu/g (*p* < 0.05) (Table 3). The counts of *B. cereus* were lower than that of the starting feed (Table 3). *Escherichia coli* counts of 4.5 log cfu/g was found on all the feeds, as well as in all the frozen larvae (Table 3). There was a 2.9 to 3.2 log reduction in the *E. coli* count on the BSFL after blanching the samples (*p* < 0.05), with *E. coli* counts ranging from 1.3–1.5 log cfu/g after blanching (Table 3).

### 3.2. Heavy Metals

Heavy metals analyzed were Al (Aluminum), As (Arsenic), Cd (Cadmium), Cr (Chromium), Fe (Iron), Hg (Mercury), Mn (Manganese), Pb (Lead), Sn (Tin) and Zn (Zinc), as these are the elements that are typically associated with health concerns and warrant in-depth investigation.

The results in Table 2 indicate that the kind of feed did not have a significant impact on Hg, Fe, Mn and Sn content and the killing method did not have a significant impact on As, Hg, Mn and Sn. The effect of feed and killing method was found to be the most prominent interaction for the heavy metals tested, which suggests that the feed the larvae are fed on, as well as killing method had a significant effect (*p* < 0.05) on the heavy metal concentration of the larvae (Table 2). The exception being Hg and Sn, where the feed and killing method interaction was not the most significant effect. The feeds analyzed all contained the heavy metals tested, except for Hg, which was not detected in any of the feeds (Table 4). There were also notable differences in the heavy metal concentrations of the three different feeds (Table 4). The feed and killing method influenced the heavy metal content, with the blanched larvae having lower concentrations of the analyzed elements with respect to frozen larvae, except for the blanched BSFL from F1 (Mn and As), and from F3 (Pb and Fe), which had higher concentrations of these elements than the frozen samples (*p* < 0.05) (Table 4). There were no significant differences between blanched and frozen larvae, with regards to certain heavy metals such as Sn, Cr (F2 and F3), Cd (F2), As (F2 and F3) and Al (F3).

Table 5 displays the bioaccumulation factor (BAF) of the nine heavy metals in BSFL from the three different feeds. Mercury has been left out of Table 5, as there was no Hg present in BSFL, therefore no bioaccumulation. The values highlighted in bold are BAF that are higher than 1, which indicated that the larvae accumulated the elements from the feed. Zinc, Mn and Cd bioaccumulated across all feed killing methods, with factors as high as 12.2 for Cadmium (BSFL from F3 frozen), 5.0 for manganese (BSFL from F2 frozen) and 1.97 for zinc (BSFL from F1 frozen) (*p* < 0.05) (Table 5). Other elements accumulated differently in the BSFL across the feed killing methods; Cr bioaccumulated in BSFL grown on F2 (blanched and frozen), As accumulated in BSFL grown on F1 and F2 (frozen), Pb accumulated in BSFL grown on F2, F3 and F1 (frozen), Fe accumulated in BSFL grown on F2 and F1 (frozen) and Sn accumulated in BSFL grown on F2 (*p* < 0.05) (Table 5). There was no accumulation of Al or Co in any of the BSFL samples (Table 5).

### 3.3. Allergen Detection

**Figure 1:**
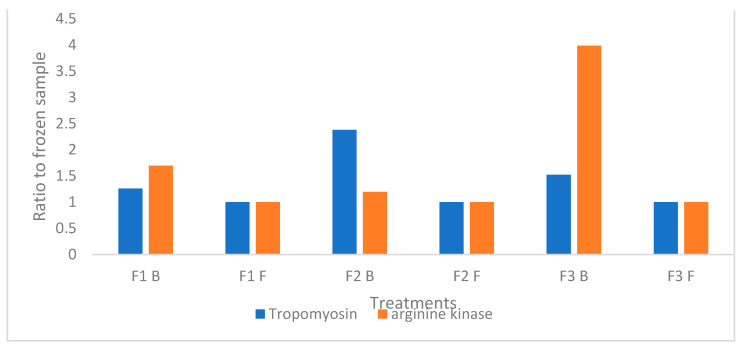
Liquid chromatography mass spectrometry (LC-MS) analysis of tropomyosin and arginine kinase in black soldier fly larvae extracts. Results represent means of three analyses and are a ratio relative to the frozen treatments. F1–Broiler-based diet; F2—Brewers grain (by-product); F3—Cereals grain (by-product); B—Blanched, F—Frozen.

One of the focuses of this study was to determine whether three main crustacean cross-reactive allergenic proteins, namely tropomyosin, arginine kinase and myosin were identified in BSFL, and whether the feed and method of killing had an influence on the allergens detected. Tropomyosin, arginine kinase and myosin peptides were identified by comparing the mass spectra from peptide fragmentation with theoretical mass spectra from in-silico digestion of the cRAP contaminant database (https://www.thegpm.org/crap/, accessed on 30 July 2021). This was carried out to identify the most similar proteins to be used as a scaffold. The Drosophila proteins were then used to generate peptide targets in the BSFL and identification of BSFL proteins was based on homology. A total of 108 different peptides relating to tropomyosin, arginine kinase and myosin were identified and after data filtering, these peptides were averaged according to the protein group. Isoforms of the same proteins were grouped together in the tables under the parental proteins, and the full table with the individual peptides and their accession numbers can be found in the addendum (Table A1). Tropomyosin had the highest average area for all the BSFL samples with abundance ranging between 82–98% in the BSFL samples, followed by arginine kinase (4–11%) and myosin (0.0–1.2%) (Table 6). Myosin was not identified in samples F2 frozen and F3 blanched and was detected in low abundance in the other BSFL samples, therefore relative abundance was not calculated for myosin. Blanching seemed to increase the detection/relative abundance of tropomyosin and arginine kinase, and for the sake of this study, results were represented by a ratio of the tropomyosin and arginine kinase relative to the frozen treatments to demonstrate the relative abundance of the proteins in each treatment (Figure 1). In blanched BSFL, tropomyosin was 1.25, 2.4 and 1.5 times higher than the frozen F1, F2 and F3 samples, respectively, and arginine kinase was 1, 1.7 and 4 times higher than the frozen samples respectively.

(Figure 1). There was variation between feed treatments, however, there was no trend to indicate that a specific feed influenced the abundance of the allergens detected.

The results of the ELISA indicated that crustacean proteins were detected in all the BSFL samples, with concentration in excess of 1600 ppm. The quantities of the crustacean allergens were all above the upper limit of the quantification method; therefore, the exact amount was not determined, and so it cannot be said whether there were specific differences between the feeds and blanched/unblanched samples.

In the development process, unique peptides to BSFL were detected (Table 6), that are not present in crustaceans and it is suggested that Multiple Reaction Monitoring (MRM) methods include these BSFL peptides to differentiate between BSFL and crustaceans when testing for allergens in an unknown food source.

## 4. Discussion

Insects are typically eaten in a processed form in the Western culture and therefore it is important to evaluate the microbial load post processing. There are currently no regulations regarding BSFL in food, however, the Netherlands Food and Consumer Product Safety Authority provided a preliminary report to assist with determining potential microorganisms that would be relevant to edible insects and suggested limits for edible insects [34]. These guidelines are largely based on EU regulations for minced meat and in the discussion of the results from this study, these regulations are used as a guideline for the microbial safety of BSFL.

Total viable count of the frozen BSFL samples (highest value at 5.47 log cfu/g) were high, although they were still considered within the regulatory limits for minced meat with regards to process hygiene criteria (<6.69 log cfu/g) (EC No 2073/2005) [33]. Total viable count and aerobic endospore formers give an indication of microbial contamination and the potential shelf-life of food products [46], therefore although the TVC count was within regulatory limits at the time of testing, it may not have a long shelf-life due to the high initial TVC counts. Blanching resulted in 3.3 to 3.4 log reductions in the BSFL TVC counts in F1, F2 and F3. The aerobic endospore former, *B. cereus*, is of particular concern as it produces heat resistant enterotoxins, which can withstand post-harvest killing methods such as blanching. Aerobic endospore formers such as *B. cereus* are recommended to not exceed log 5 cfu/g in edible insects, and in the case of the frozen (2–2.17 log cfu/g) and blanched (1.69–2 log cfu/g) BSFL the levels of aerobic endospore formers are well below the recommended limits [34]. These values are similar to previous studies which found BSFL to contain quantities of 2.3 log cfu/g of *B. cereus* [7]. Aerobic endospore formers present a challenge for edible insects [12,47,48,49] due to the difficulty in irradicating them, therefore it is suggested that strategies be adopted to use feed with low aerobic spore counts to limit possible contamination [50]. The results from this study did not demonstrate *B. cereus* accumulation in the BSFL, as the BSFL had lower *B. cereus* than the feeds they were grown on. This was also observed in a study where *B. cereus* in the BSFL did not accumulate from the feed [7].

Indicator organisms such as, *Escherichia coli,* are usually tested in foods to determine potential food hygiene. *Escherichia coli* ranged from 1.30 log cfu/g in the blanched samples to 4.47 log cfu/g in the frozen samples. The blanched samples were below regulations for minced meat (2.69 log cfu/g) however, the frozen samples exceeded the regulatory limits for *E. coli* in minced meat with regards to process hygiene criteria (EC No 2073/2005) [33]. *Escherichia coli* was also found in high numbers in other fresh, untreated insect species such as the African rhinoceros beetle (*Oryctes monocerus*) [8], mealworms (*Tenebrio molitor*) and crickets (*Acheta domesticus*) [12], indicating that *E. coli* should be tested for in edible insects to ensure food safety.

Both *Salmonella* and *L. monocytogenes* were not detected in 25 g of the feed or the BSFL samples (frozen and blanched). This complies with EU standards for minced meat and meat preparations intended to be eaten raw or cooked (EC No 2073/2005) [33]. *Salmonella* and *L. monocytogenes* are not typically found in BSFL [7], however, *Salmonella* and *L. monocytogenes* are common food-borne pathogens that are required to be tested for in high moisture foods (EC No 2073/2005) [33], and due to the high-risk nature of these pathogens, it is worth ensuring neither is present in BSFL samples destined for human food. Furthermore, if *Listeria* spp. are present in the feed in high concentrations, it will ultimately reflect in the BSFL [13], therefore it is important to also test for *Listeria* spp. in BSFL as it can be present if the feed is contaminated, as well as during processing. Even though BSFL have demonstrated the ability to significantly reduce Enterobacteriaceae colonies and *Salmonella* spp. in their feed [2,14,15], this study still showed high *E. coli* counts on the frozen larvae that reflected the *E. coli* count on the feed, therefore it is worth ensuring there are adequate post-harvesting hurdles such as blanching to reduce the microbial count to within safe limits.

As mentioned previously, studies have determined that the feed influences the microbial load of BSFL [51,52,53,54], however, it has been suggested that the situation is more complex and there are a multitude of factors at play that could affect the resulting microbial load of BSFL [7]. Feed is one of them, as well the rearing method, amount of handling, interaction with other microbial communities and the parental origin of the BSFL [7]. This study indicated that the feed only influenced the endospore formers, however, the feed did not have an effect on the *E. coli* nor the TVC of the BSFL. The killing method was found to have more defined effect on the microbial content of the larvae. Blanching has been found to be the most effective and economically viable decontamination tool and it has been recommended that it be employed in edible insect production to ensure food safety of edible insects [12,34,47,48]. This study found blanching to reduce microbial loads in BSFL to within safe limits for human consumption, further validating the recommendation to blanch edible insects as a post-harvesting killing method. Blanching was not able to reduce the endospore formers, although their counts was below legislated limits. Another study on BSFL confirmed these findings that blanching is an effective tool in reducing microbial content of BSFL [49]. Alternative decontamination methods have been proposed such a high-pressure pasteurizing (HPP) however, studies have found it less effective than blanching [49,55]. Blanching remains the best commercial tool available to reduce the microbial load of BSFL, although special consideration would need to be placed on the endospore forming bacteria to ensure they remain below legislated limits [12,49,55,56,57,58]. Technologies, such as high-pressure pasteurizing in combination with a heat treatment can be effective in reducing bacterial spores, although it typically has poor consequences on the organoleptic properties of the food [56]. More novel approaches include using natural bioactive substances in the feed of the BSFL to potentially control microbial pathogens [59,60].

The substrate used to feed the larvae and the killing method clearly influenced the concentration of the mineral elements analyzed. Previous studies have also found the feed to have an influence on the heavy metal content of the larvae [17,18,19,20]. The frozen samples generally had higher concentrations than the blanched samples, and it is speculated that this is because some of the elements may have leached into the blanching water, similarly to what occurs when blanching vegetables [57,58]. Further research is suggested to determine exactly how much leaching occurs during blanching and how blanching time effects the various heavy metal concentrations. There were exceptions to this observation (Mn-F1, Fe-F3; Pb-F3; As-F1), although the exact cause for the latter exceptions is unclear.

Mercury (Hg), one of the most toxic heavy metals is known for causing mercury poisoning when highly contaminated fish were consumed [61,62]. Mercury levels were not detected in most samples (LOQ = 1 µg/kg), except for BSFL from F2 (frozen) and F3 (frozen); nonetheless, they were both well below legislated limits required by the European Commission for Hg in fish (0.5 mg/kg) (EC 1881/2006 and amending regulations) [63]. There was no bioaccumulation of Hg in this study, most probably because there was no Hg detected in the feed samples, although this is not to say that Hg is not accumulated when it is present in the feed. Mercury typically bioaccumulates in BSFL, with a BAF range from 1.6 [17] to 4.5 [18] depending on the feed. Even though bioaccumulation of Hg is a concern, studies have found that even when there is bioaccumulation of Hg, the Hg concentrations are still well below legal limits in feed and food [17,18]. Cadmium (Cd) is another highly toxic, carcinogenic heavy metal that has serious health concerns for humans when exposed [62]. The Cd concentrations in the BSFL in this study (0.11–0.17 mg/kg) were slightly lower than previous studies (0.19–1.4 mg/kg), which could be attributed to the lower Cd concentration of the feeds in this study [17,18,19]. The BAF of Cd (4.67–12.27) in this study was comparable to previous studies (2.5–9.5) [18,19], indicating that Cd is highly accumulative, although the Cd bioaccumulation of the frozen BSFL grown on F3 (12.27) was twice as high as previous studies. Black soldier fly larvae contain an abundance of Calcium channels in their gut, which facilitate the uptake of Cadmium resulting in high Cd bioaccumulation comparatively to other heavy metals [6,63]. Arsenic (As) is essential in small doses, however, in excess it is poisonous and carcinogenic [62]. The As values in this study ranged from 0.03–0.15 mg/kg, which were similar to a previous study with concentrations between 0.12–0.13 mg/kg [18], but much lower than other studies where BSFL had concentrations of 2 mg/kg [19] and 2.1–3.3 mg/kg [17]. Arsenic bioaccumulated in some samples (BSFL fed F1 blanched and frozen; BSFL fed F2 frozen), but not in others. These inconsistencies were also found, where some BSFL bioaccumulated As from certain feeds, but not from other feed [19] whilst in other studies As was found to not accumulate at all [18]. Further studies should investigate factors affecting bioaccumulation, as this would give insight into why it accumulates from certain feed and not from others. Lead (Pb) concentrations ranged from 0.13–0.26 mg/kg which was consistent with previous findings [17,20]. Bioaccumulation of Pb occurred in all but one sample (BSFL fed F1 blanched), with BAF ranges of 1.11–3.2, which could have possibly leached out upon blanching. Previous studies had similar BAF ranges from 1.1 [19] to 2.3 [18]. Tin (Sn) has low toxicity, due to limited absorption in the gut, however, over consumption can lead to nausea and muscular weakness [61]. The levels of Sn were low (0.01–0.04 mg/kg), especially when considering the regulations allow up to 50 mg/kg of Sn in a product before it is considered hazardous (EC 1881/2006) [63]. There was bioaccumulation of Sn only in the larvae grown on F2 (blanched and frozen).

The heavy metals in question were analyzed to determine whether consuming BSFL would be a safety concern for humans. Whilst there are no specific regulations pertaining to the limits for BSFL for human consumption, the European Commission’s guidelines for contaminants in crustaceans, fish, meat and mealworms were used as a benchmark for the safety of BSFL for human consumption. According to the regulations set out for crustaceans and seafood, the heavy metal content in BSFL was below maximum limits with regards to the legislated contaminants As, Cd, Sn, Hg and Pb (EC 1881/2006) [63]. Regulatory limits are stipulated for wet weight, and while the heavy metals in this study were documented on a dry matter basis, it would still fall below legislated limits if consumed in a wet form. When using the maximum levels for meat as a benchmark (Cd > 0.05 mg/kg; Pb > 0.1), then all the BSFL samples exceeded the maximum limits for both Cd and Pb (EC 1881/2006) [63]. The same would be true if the maximum limits recently set out for mealworms for human consumption was used as the benchmark (Cd > 0.1 mg/kg; Pb > 0.075) [62]. This clearly demonstrates that further investigations are needed to set limits that would be considered more appropriate for BSFL [18]. These investigations should include rigorous studies determining the effect of different feeds on the heavy metal content of BSFL. This is substantiated by a study that demonstrated that mealworms and BSFL have different innate heavy metal contents, and bioaccumulate heavy metals differently [19], therefore it would not be recommended to use the mealworm guidelines for BSFL, as they are innately different insect species in terms of their mineral content. While these legislated heavy metals are an important safety concern, only certain elements are considered contaminants and have legislated limits in foods, among them are the previously discussed As, Cd, Sn, Hg and Pb (EC 1881/2006) [63]. This is however not an exhaustive list of the heavy metals that can cause health concerns; Al, Cr, Fe, Mn and Zn, have also been known to cause toxicity when consumed at high concentrations and are worth discussing further.

Aluminum (Al) is found in trace amounts in various foods without causing concern, however, there are cautions set out by the Food and Agricultural Organization (FAO) and the World Health Organization (WHO) to prevent the bioaccumulation of Al, which has the potential to negatively affect the reproductive and nervous system [64]. A weekly recommended limit of 2 mg/kg bodyweight is suggested, indicating that a 60 kg adult could safely consume 120 mg/kg of Al per week [64] and the Al concentration in BSFL (17.59 mg/kg–52 mg/kg) is well within those recommended limits as a human would need to consume ≈ 3 kg of BSFL per week. Manganese (Mn) is essential to humans, and although oral ingestion has low toxicity, it can cause neurotoxicity at high concentrations [63,64]. Manganese (Mn) concentrations in the larvae ranged from 454.16 mg/kg to 525.69 mg/kg which is higher than a previous study that found Mn concentrations of 200 mg/kg in BSFL [17]. Iron (Fe) is essential in the human body however, humans do not have a mechanism to maintain homeostasis for Fe, and it can therefore accumulate in the blood stream and become toxic [64]. The range of Fe in the BSFL in this study (144.6 mg/kg–252.7 mg/kg) was lower than noted previously where Fe contents ranged from 500 mg/kg to 600 mg/kg [17]. It is higher than Fe values typically found in red meat (11–20 mg/kg) [65]. Chromium, another essential element in the diet, can be toxic when consumed at levels above 200 mg at a time [66], but the Cr contents in the BSFL were too low to cause concern with Cr ranges of 0.22 mg/kg to 0.52 mg/kg. These values are slightly higher than found in BSFL grown on uncontaminated substrate (0.064 mg/kg) and lower than BSFL grown on contaminated feeds, with Cr concentrations of 3.4 mg/kg [60] and 16.52 mg/kg [67]. There was low bioaccumulation of Cr in the larvae grown on F2, and no bioaccumulation on F1 and F3, although previous findings have not found Cr to bioaccumulate in BSFL [67]. Zinc’s daily requirement is between 5–11 mg/d and although Zn is not highly toxic, overconsumption can cause decreased absorption of other minerals such as Fe [68]. This may be of concern as BSFL was high in Zn (129.7 mg/kg–220.6 mg/kg), which is consistent with previous findings (146 mg/kg–285 mg/kg) [8].

When comparing the results from this study with previous studies, it is evident that there are differences in heavy metals in BSFL across studies, which can be attributed to varying heavy metal content as BSFL age [18,63] and to feed. It is therefore it is important to ensure that the feed used for BSFL contain low levels of heavy metals to reduce the risk of bioaccumulation to levels that may potentially cause risk to consumers. It will be critical to introduce mitigation tools to prevent the uptake of contaminants from the feed. HAACP is a powerful tool that can assist with identifying risks and mitigating against them by applying appropriate processing steps, and far more studies such as this one is required to gather information to develop a robust HACCP protocol [35]. It is also worth mentioning, that aside from the health concerns associated with high concentrations of heavy metals in BSFL, high concentrations are also detrimental to the counts of BSFL themselves and therefore it is important for the heavy metals to be managed both for the health of the larvae and of those who consume them [6,21].

Tropomyosin, and arginine kinase were detected in high abundance in all BSFL samples using mass spectrometry. Myosin, on the other hand was detected in low abundance, and was not found in two of the BSFL samples (F2 frozen and F3 blanched). Proteomics, specifically mass spectrometry (MS), has been increasingly used as a tool to accurately identify specific allergenic proteins in foods. There are many benefits of using MS for allergen detection, with the main one being that it can detect allergens at low concentrations and equally at high concentrations [59]. Tropomyosin and arginine kinase are common cross-reactive allergens found in edible insects [25,26,27,31,60], and both have been detected in BSFL in a previous study [27]. Another study on BSFL, only detected tropomyosin, whereas arginine kinase and myosin were not detected at all [24]. Blanched proteins were in higher abundance in all the BSFL samples, which is a phenomenon that has been observed in previous studies; these studies attribute the higher abundance of detectable allergic proteins to an increase in extractable proteins and increased protein solubility after heating [24,27]. This is speculated to occur because of the changes in the protein structure after heat treatments [24,27]. Even though the results indicate a higher abundance of these proteins, the clinical significance would need to be investigated, as has been noted where the allergenic proteins increased after heating, although skin prick tests did not yield conclusive findings that heat processed samples increased the reaction to the allergens [27]. Additionally, unique peptides were identified in BSFL that could be used in MRM methods to differentiate between BSFL and crustaceans in a food source.

Commercially, the antibody-based enzyme-linked immunosorbent assay (ELISA) kits are used to determine the presence of crustacean allergens, however, one of the challenges with ELISA is that although the kits are designed to target crustacean allergens, false positive results have occurred due to cross-reactivity [60]. This was found to be the case in this study, as the EILSA tests showed presence of crustacean proteins in high concentrations (>1600 mg/kg) in the BSFL samples. Black soldier fly larvae were reared in controlled environments and had no contact with crustaceans, therefore the results from the EILSA are clearly demonstrating cross-reactivity. Based on the ELISA results, consumers with crustacean allergies would have an allergic reaction when consuming BSFL, especially considering the allergen dosage is recommended at 25 mg/kg (http://allergenbureau.net/vital, accessed on 30 July 2021) where anything above that would cause an allergic reaction. The quantity of the crustacean allergens was above the upper limit of the quantification method; therefore, the exact amount was not determined, and it cannot be said whether there were specific differences between the feeds and blanched/unblanched samples. Although this could be considered helpful for potential consumers who are allergic to crustacean, the results are technically inaccurate, as there are no crustaceans proteins in the BSFL samples. There is a difference between clinical and analytical cross-reactivity. It is not necessarily true that allergic consumers would react.

## 5. Conclusions

This investigation set out to determine whether the feed the BSFL are fed on and killing method (blanching or freezing) would influence the bacterial load on, and the accumulation of heavy metals—ultimately affecting the food safety of BSFL. It was established that the killing method had a significant influence on the microbial load and the heavy metal content of the BSFL; as expected blanching significantly reduced microbial contamination of the BSFL, as well as resulted in slightly lower heavy metal concentrations in the larvae. The feed fed to the larvae was also found to influence the heavy metal content of the BSFL, with heavy metals such as Zn, Mn, Cr, Pb, Fe, Sn and Cd bioaccumulating. Using seafood regulations as a benchmark, BSFL were below legal limits for all potential risky heavy metals, and the blanched larvae were also considered safe to eat according to the EU regulations for microbial limits in minced meat. Furthermore, this study identified the presence of three main cross-reactive allergens, namely tropomyosin, arginine kinase and myosin in the BSFL samples through mass spectrometry, with the exception of myosin not being detected in two samples (F2 frozen and F3 blanched). Feed did not seem to influence the abundance of the allergens, however, blanching increased the relative abundance of tropomyosin and arginine kinase, although the clinical relevance of this still needs to be established. A commercial ELISA kit confirmed analytical cross-reactivity by detecting crustacean allergens in the BSFL samples. Overall, blanching is strongly suggested to reduce the microbial load, as well as the heavy metal content and should be implemented to ensure the food safety of BSFL.

## Figures and Tables

**Table 1:** Ingredient formulation for three different black soldier fly larvae diets.

F1 Broiler-Based Diet	F2 Brewers’ Grain (By-Product)	F3 Cereal Grains (By-Product)
5.5 kg wheat bran	9 kg brewers’ grain	3 kg maize
5.5 kg broiler pellets	1 kg brewers’ yeast	3 kg soya
0.06 kg micropack	1 kg wheat bran	3 kg wheat bran
21 kg water	0.06 kg micropack	2 kg oat bran
	21 kg water	0.06 kg micropack
		21 kg water

F1—Feed 1; F2—Feed 2; F3—Feed 3. Micropack (phosphorus, calcium, potassium).

**Table 2:** The *p*-values indicating the impact of feed and treatment on the various heavy metals and microbial counts measured for black soldier fly larvae.

	Feed	Killing Method	Feed X Killing Method
Total viable count	0.531	0.000	0.531
*Bacillus cereus*	0.001	0.123	0.011
*Escherichia coli*	0.771	0.000	0.771
Al	0.005	<0.001	<0.001
Cr	0.004	0.002	0.002
As	0.003	0.178	0.019
Cd	0.004	<0.001	0.002
Hg	0.170	0.097	0.170
Pb	<0.001	<0.001	<0.001
Fe	0.983	<0.001	<0.001
Mn	0.940	0.631	0.019
Sn	0.792	0.156	0.526
Zn	<0.001	<0.001	<0.001

Al (Aluminium), As (Arsenic), Cd (Cadmium), Cr (Chromium), Fe (Iron), Hg (Mercury), Mn (Manganese), Pb (Lead), Sn (Tin), Zn (Zinc).

**Table 3:** Microbial results of feed and black soldier fly larvae grown on three different feeds after being frozen and blanched (log cfu/g).

	F1	F2	F3	BSFL F1 B	BSFL F1 F	BSFL F2 B	BSFL F2 F	BSFL F3 B	BSFL F3 F
Total viable count	TNTC	TNTC	TNTC	2.1 ^b^ ± 0.3	5.5 ^a^ ± 0.4	2.2 ^b^ ± 0.00	5.5 ^a^ ± 0.9	2.2 ^b^ ± 0.1	5.5 ^a^ ± 0.8
*Bacillus cereus*	5.5 ± 0.5	5.5 ± 0.6	5.5 ± 0.5	1.7 ^c^ ± 0.0	2.0 ^b^ ± 0.01	2.0 ^b^ ± 0.2	2.2 ^c^ ± 0.0	2.0 ^b^ ±0.2	2.0 ^b^ ± 0.2
*Escherichia coli*	4.5 ± 0.00	4.5 ± 0.1	4.5 ± 0.00	1.5 ^b^ ± 0.8	4.5 ^a^ ± 0.5	1.5 ^b^ ± 0.00	4.5 ^a^ ± 0.5	1.3 ^b^ ± 0.8	4.5 ^a^ ± 0.00
*Salmonella* spp.	ND	ND	ND	ND	ND	ND	ND	ND	ND
*Listeria* spp.	ND	ND	ND	ND	ND	ND	ND	ND	ND

ND—None detected; TNTC—too numerous to count. F1 = Feed 1 Broiler-based; F2 = Feed 2 Brewers grain; F3 = Feed 3 Cereal grain; B—Blanched, F—Frozen. ^a–c^ Means of treatments with different superscripts in each row differ significantly (*p* < 0.05).

**Table 4:** Heavy metal content (mg/kg dry matter basis) of black soldier fly larvae grown on three different feeds and killed by blanching and freezing (means and SD).

	F1	F2	F3	BSFL F1 B	BSFL F1 F	BSFL F2 B	BSFL F2 F	BSFL F3 B	BSFL F3 F
Al	65.24 ± 0.02	60.75 ± 0.03	59.97 ± 0.04	17.59 ^c^ ± 0.81	52.00 ^a^ ± 0.06	20.25 ^c^ ± 1.2	28.16 ^b^ ± 1.17	27.96 ^b^ ± 1.09	32.16 ^b^ ± 0.08
Cr	0.65 ± 0.12	0.37 ± 0.92	2.99 ± 0.32	0.22 ^c^ ± 0.04	0.52 ^a^ ± 0.02	0.46 ^a^ ± 0.03	0.47 ^a^ ± 0.05	0.31 ^b^ ± 0.04	0.33 ^b^ ± 0.03
As	0.05 ± 0.25	0.05 ±0.02	0.00 ±0.01	0.15 ^b^ ± 0.01	0.12 ^a^ ± 0.01	0.02 ^c^ ± 0.01	0.06 ^c^ ± 0.01	0.03 ^b^ ± 0.01	0.03 ^b^ ± 0.01
Cd	0.02 ± 0.01	0.02 ± 0.01	0.01 ± 0.12	0.11 ^b^ ± 0.01	0.17 ^a^ ± 0.01	0.12 ^b^ ± 0.01	0.12 ^b^ ± 0.01	0.11 ^a^ ± 0.01	0.17 ^b^ ± 0.01
Hg	<DL	<DL	<DL	<DL	<DL	<DL	<DL	<DL	<DL
Pb	0.14 ± 0.01	0.08 ± 0.01	0.12 ± 0.01	0.13 ^e^ ± 0.01	0.18 ^c^ ± 0.01	0.23 ^b^ ± 0.01	0.26 ^a^ ± 0.01	0.16 ^d^ ± 0.01	0.14 ^e^ ± 0.01
Fe	244.0 ± 0.7	146.0 ± 0.8	258.0 ± 0.5	144.7 ^d^ ± 0.4	252.7 ^a^ ± 0.1	185.4 ^c^ ± 0.2	210.6 ^b^± 0.6	201.2 ^bc^ ± 0.9	197.3 ^bc^ ± 0.3
Mn	165.0 ± 0.3	99.0 ± 0.7	107.0 ± 0.6	525.7 ^a^ ± 0.1	454.2 ^c^ ± 0.7	490.0 ^abc^ ± 0.1	493.9 ^abc^ ± 0.3	470.8 ^bc^ ± 0.9	519.7 ^ab^ ± 0.8
Sn	0.14 ± 0.01	0.01 ± 0.01	0.24 ± 0.01	0.01 ± 0.02	0.03 ± 0.01	0.02 ± 0.01	0.02 ± 0.01	0.04 ± 0.01	0.02 ± 0.01
Zn	112.0 ± 0.65	90.0 ± 0.22	108.0 ± 0.13	129.8 ^d^ ± 0.35	220.6 ^a^ ± 0.23	134.0 ^d^ ± 0.15	139.7 ^cd^ ± 0.93	146.6 ^c^ ± 0.71	166.8 ^b^ ± 0.10

^a–e^ Means with different superscripts within rows differ significantly (*p* < 0.05). Al (Aluminum), As (Arsenic), Cd (Cadmium), Cr (Chromium), Fe (Iron), Hg (Mercury), Mn (Manganese), Pb (Lead), Sn (Tin), Zn (Zinc). F1 = Feed 1 Broiler-based; F2 = Feed 2 Brewers grain; F3 = Feed 3 Cereal grain; B—Blanched, F—Frozen. Detection Limit (DL) for Hg = 1 µg/kg.

**Table 5:** The bioaccumulation factor (means and SD) for select heavy metals for BSFL grown on three different feeds and killed by freezing or blanching (*p* < 0.05).

	BSFL F1 B	BSFL F1 F	BSFL F2 B	BSFL F2 F	BSFL F3 B	BSFL F3 F
Al	0.2 ± 0.46	0.8 ± 0.00	0.3 ± 0.56	0.4 ± 0.15	0.4 ±0.13	0.5 ± 0.19
Cr	0.3 ± 0.29	0.8 ± 0.00	1.2 ± 0.01	1.2 ± 0.00	0.1 ± 0.01	0.1 ± 0.00
Co	0.4 ± 0.24	0.5 ± 0.00	0.5 ± 0.00	0.5 ± 0.00	0.5 ± 0.00	0.3 ± 0.00
As	3.0 ± 0.45	2.4 ± 0.24	0.3 ± 0.38	1.1 ± 0.76	0.1 ± 0.24	0.1 ± 0.31
Cd	4.6 ± 0.08	7.0 ± 0.25	6.7 ± 0.53	6.9 ± 0.35	8.2 ± 0.26	12.2 ± 0.03
Pb	0.8 ± 0.12	1.2 ± 0.48	2.8 ± 0.09	3.2 ± 0.04	1.2 ± 0.01	1.1 ± 0.02
Fe	0.5 ± 0.02	1.0 ± 0.36	1.2 ± 0.14	1.4 ± 0.02	0.7 ± 0.01	0.7 ± 0.01
Mn	3.1 ± 0.04	2.7 ± 0.03	4.9 ± 0.45	5.0 ± 0.16	4.4 ± 0.03	4.8 ± 0.49
Sn	0.1 ± 0.01	0.2 ± 0.69	2.1 ± 0.09	2.3 ± 0.92	0.1 ± 0.18	0.1 ± 0.06
Zn	1.1 ± 0.040	1.9 ± 0.3	1.4 ± 0.09	1.5 ± 0.01	1.3 ± 0.02	1.5 ± 0.13

Values highlighted in bold are higher than 1 and considered heavy metals that have been accumulated from the feed. Al (Aluminum), As (Arsenic), Cd (Cadmium), Cr (Chromium), Fe (Iron), Hg (Mercury), Mn (Manganese), Pb (Lead), Sn (Tin), Zn (Zinc). F1 = Feed 1 Broiler-based; F2 = Feed 2 Brewers grain; F3 = Feed 3 Cereal grain; B—Blanched, F—Frozen.

**Table 6:** Unique proteins and accession numbers identified in black solider fly larvae to be used to differentiate between black soldier fly larvae and crustaceans.

Protein	Accession Number	Peptide Sequence	Position
Myosin	Q98323	ALESQLAELK	1620 to 1629
Arginine Kinase	P48610	VSSTLSGLEGELK	152 to 164
Arginine Kinase	P48610	LEEGYAK	10 to 16
Tropomyosin	P06754	FLAEEADK	207 to 214
Tropomyosin	P49455	ALQNAESEVAALNR	77 to 90
Tropomyosin	P06754	LEDDLVLEK	310 to 318

## Data Availability

The data presented in this study are available on request from the corresponding author.

## Appendix A

**Table A1:** Three common cross-reactive crustacean allergenic peptides which presented a positive hit in the liquid chromatography mass spectrometry (LS-MS). The in-silico assessment was carried out using cRAP contaminant database on black soldier fly peptides identified.

	**Component Name**	**Sum of Peaks**
Feed 1 Blanched	Tropomyosin.FLAEEADKy3	2478
	Arginine Kinase.LEEGYAKy3	12,300.6
	Tropomyosin.ALQNAESEVAALNRy4	103,009
	Tropomyosin.SLEVSEEK.y4	566,441
	Tropomyosin.SLEVSEEKy3	21,440
	Myosin.ALESQLAELKy4	719.2
	Arginine Kinase.LEEGYAKy4	1036.7
	Arginine Kinase.VSSTLSGLEGELKy4	12,976.2
	Tropomyosin.FLAEEADKy3	2102
	Tropomyosin.ALQNAESEVAALNRy4	104,538
	Tropomyosin.LEDDLVLEKy4	652.9
	Tropomyosin.SLEVSEEK.y4	583,612
	Tropomyosin.SLEVSEEKy3	21,614
Feed 1 Frozen	Myosin.ALESQLAELKy4	589.9
	Myosin.ALESQLAELKy4	1592
	Arginine Kinase.LEEGYAKy4	9136
	Arginine Kinase.VSSTLSGLEGELKy4	1804
	Tropomyosin.FLAEEADKy3	3817
	Tropomyosin.ALQNAESEVAALNRy4	16,776
	Tropomyosin.LEDDLVLEKy4	6878
	Tropomyosin.SLEVSEEK.y4	578,595
	Tropomyosin.SLEVSEEKy3	22,069.7
	Arginine Kinase.LEEGYAKy4	8642
	Arginine Kinase.VSSTLSGLEGELKy4	1125
	Tropomyosin.FLAEEADKy3	4197.8
	Tropomyosin.ALQNAESEVAALNRy4	17,089
	Tropomyosin.LEDDLVLEKy4	6695.4
	Tropomyosin.SLEVSEEK.y4	571,687
	Tropomyosin.SLEVSEEKy3	23,102.6
Feed 2 Blanched	Arginine Kinase.LEEGYAKy4	773.5
	Arginine Kinase.VSSTLSGLEGELKy4	6243
	Tropomyosin.FLAEEADKy3	554.3
	Tropomyosin.ALQNAESEVAALNRy4	33,908
	Tropomyosin.SLEVSEEK.y4	120,925.3
	Tropomyosin.SLEVSEEKy3	4455
	Myosin.ALESQLAELKy4	520.3
	Arginine Kinase.LEEGYAKy4	454.4
	Arginine Kinase.VSSTLSGLEGELKy4	8471.8
	Tropomyosin.FLAEEADKy3	543.1
	Tropomyosin.ALQNAESEVAALNRy4	5808
	Tropomyosin.SLEVSEEK.y4	116,240.1
	Tropomyosin.SLEVSEEKy3	4257
Feed 2 Frozen	Arginine Kinase.LEEGYAKy4	4498.2
	Arginine Kinase.VSSTLSGLEGELKy4	2123
	Tropomyosin.FLAEEADKy3	4914.5
	Tropomyosin.ALQNAESEVAALNRy4	16,397
	Tropomyosin.LEDDLVLEKy4	7620.7
	Tropomyosin.SLEVSEEK.y4	788,592
	Tropomyosin.SLEVSEEKy3	27,783.7
	Arginine Kinase.LEEGYAKy4	4653.4
	Arginine Kinase.VSSTLSGLEGELKy4	2069
	Tropomyosin.FLAEEADKy3	5162
	Tropomyosin.ALQNAESEVAALNRy4	17,179
	Tropomyosin.LEDDLVLEKy4	8624.8
	Tropomyosin.SLEVSEEK.y4	785,594
	Tropomyosin.SLEVSEEKy3	27,579
Feed 3 Blanched	Arginine Kinase.LEEGYAKy4	1155.8
	Arginine Kinase.VSSTLSGLEGELKy4	22,693
	Tropomyosin.FLAEEADKy3	1302
	Tropomyosin.ALQNAESEVAALNRy4	60,192
	Tropomyosin.LEDDLVLEKy4	902.3
	Tropomyosin.SLEVSEEK.y4	386,547
	Tropomyosin.SLEVSEEKy3	13,167.1
	Arginine Kinase.LEEGYAKy4	859.7
	Arginine Kinase.VSSTLSGLEGELKy4	18,880.9
	Tropomyosin.FLAEEADKy3	2045.8
	Tropomyosin.ALQNAESEVAALNRy4	10,642
	Tropomyosin.LEDDLVLEKy4	839
	Tropomyosin.SLEVSEEK.y4	393,257
	Tropomyosin.SLEVSEEKy3	15,912.3
Feed 3 Frozen	Myosin.ALESQLAELKy4	710.1
	Arginine Kinase.LEEGYAKy4	5035
	Arginine Kinase.VSSTLSGLEGELKy4	478.9
	Tropomyosin.FLAEEADKy3	481.1
	Tropomyosin.ALQNAESEVAALNRy4	9495
	Tropomyosin.LEDDLVLEKy4	2755.2
	Tropomyosin.SLEVSEEK.y4	264,799.3
	Tropomyosin.SLEVSEEKy3	9668.8
	Arginine Kinase.LEEGYAKy4	4867.9
	Arginine Kinase.VSSTLSGLEGELKy4	553.2
	Tropomyosin.FLAEEADKy3	2249
	Tropomyosin.ALQNAESEVAALNRy4	8680
	Tropomyosin.LEDDLVLEKy4	4034
	Tropomyosin.SLEVSEEK.y4	266,701.5
	Tropomyosin.SLEVSEEKy3	11,924
